# Decision support for augmented reality-based assistance systems deployment in industrial settings

**DOI:** 10.1007/s11042-024-19861-x

**Published:** 2024-08-13

**Authors:** Lukas Bock, Thomas Bohné, Sławomir K. Tadeja

**Affiliations:** 1 Department of Engineering, University of Cambridge, Cambridge, CB2 1PZ UK; 2 School of Engineering and Design, Technical University of Munich, 80333 Munich, Germany

**Keywords:** Augmented Reality, AR, Decision Support, Customised Deployment Strategies, Human-Centric Manufacturing

## Abstract

The successful deployment of augmented reality (AR) in the industry for on-the-job guidance depends heavily on factors such as the availability of required expertise, existing digital content and other deployment-related criteria such as a task’s error-proneness or complexity. Particularly in idiosyncratic manufacturing situations involving customised products and diverse complex and non-complex products and its variants, the applicability and attractiveness of AR as a worker assistance system is often unclear and difficult to gauge for decision-makers. To address this gap, we developed a decision support tool to help prepare customised deployment strategies for AR-based assistance systems utilising manual assembly as the main example. Consequently, we report results from an interview study with sixteen domain experts. Furthermore, when analysing captured expert knowledge, we found significant differences in criteria weighting based on task complexity and other factors, such as the effort required to obtain data.

## Introduction

Manufacturing is undergoing significant changes that demand increased efficiency and effectiveness while simultaneously dealing with growing supply and complex production processes [1]. For example, modern production companies operate in volatile environments or face rapidly changing customer preferences, and the demand for customisation requires manufacturers to produce in small batches and frequently adjust their production [2]. Consequently, companies have to reconsider their manufacturing strategies to factor in increasing quality requirements and rapidly shortened innovation and development cycles [3, 4].

Although the automation of production lines has become more advanced, human workers also continue to play an essential role in manufacturing [5, 6]. Companies need workers who are able to develop new products and simultaneously troubleshoot, control, and perform complex operations. Workers need to react independently to unforeseen changes based on their knowledge and experience to provide flexibility and responsiveness on the given manufacturing shop floor [7]. Developing such skills among workers is even more essential due to the growing elasticity of production and the relatively high personnel turnover for simple tasks [8]. Thus, affordable on-boarding and on-the-job guiding approaches for novice workers in new or changed production processes are critically important. Traditional guiding methods to address this requirement, such as personal demonstration, videos or paper manuals, are limited by their cost or flexibility to changes. Frequently evolving production processes require rapid adaptation to changing circumstances, which tends to reduce efficiency and increase workers’ cognitive load. There is, therefore, a high and growing need for adaptable and continuous employee training to keep up with the frequent changes in manufacturing processes [9]. Frequently evolving production processes require rapid adaptation to changing circumstances, which tends to reduce efficiency and increase workers’ cognitive load. There is, therefore, a high and growing need for adaptable and continuous employee training to keep up with the frequent changes in manufacturing processes [9].

**Fig. 1:**
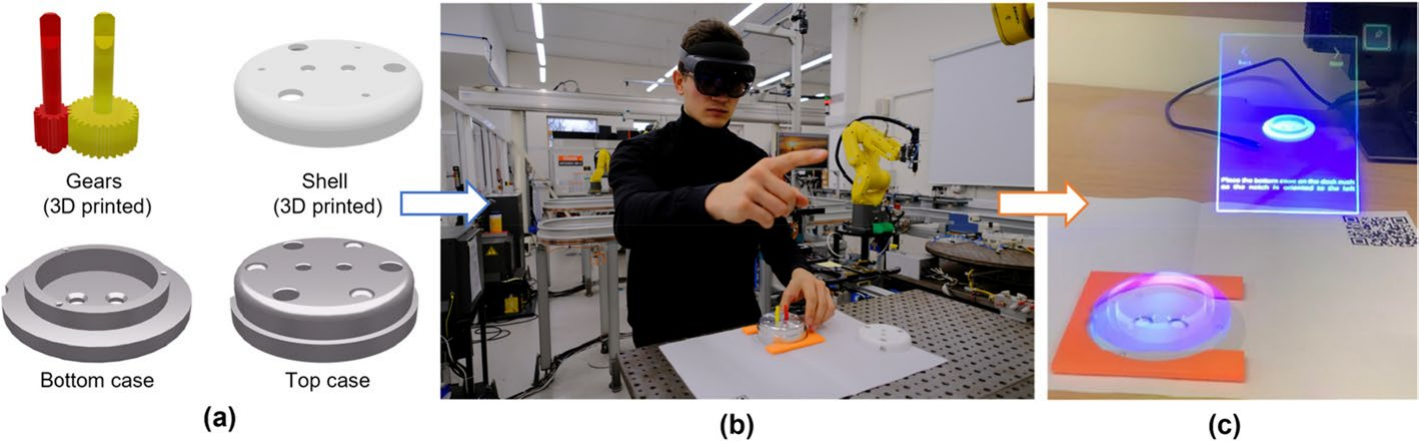
(**a**) An example of an engineering asset requiring manual assembly–an eight-part gearbox. (**b**) A novice shop floor worker using the augmented reality (AR) head-mounted display to guide the manual assembly process of a low-complexity gearbox object. (**c**) The view through the AR headset as seen by the shop floor worker during the manual assembly process

In that context, augmentation technology is one type of assistance system gaining more attention in the industry. The term “augmentation technology” serves as an umbrella term for various technologies, including cognitive augmentation with, for example, AR [10] and physical augmentation with exoskeletons, that can improve information delivery and task performance of workers to enhance their work [7]. These state-of-the-art assistance solutions, especially AR technology, have the potential to address industrial needs concerning adaptation to changing manufacturing processes (see Fig. 1) [10–12].

Certain manufacturing tasks can induce a high cognitive load, leading to inefficiencies and errors. AR addresses this by overlaying contextual, visual information, thus reducing cognitive load and increasing productivity, as demonstrated by [36]. Furthermore, manual assembly and training processes are often time-consuming and error-prone. Here, AR can streamline these processes by providing interactive instructions directly on workpieces, significantly reducing learning times and improving accuracy [34]. This direct support in addressing the complexity and skills gaps in manufacturing underscores the value of AR [37].

However, for these systems to be valuable tools and useful on a factory’s shop floor, it is important to understand factors related to their deployment and life cycle. This includes design, development and deployment-related costs, as well as their net value to the manufacturing processes. Thus, it is crucial to comprehend the idea of assisting employees in their tasks and to investigate the possible advantages of deploying cutting-edge technologies, e.g. AR-based systems, on an actual manufacturing shop floor [10–14].

**Fig. 2:**
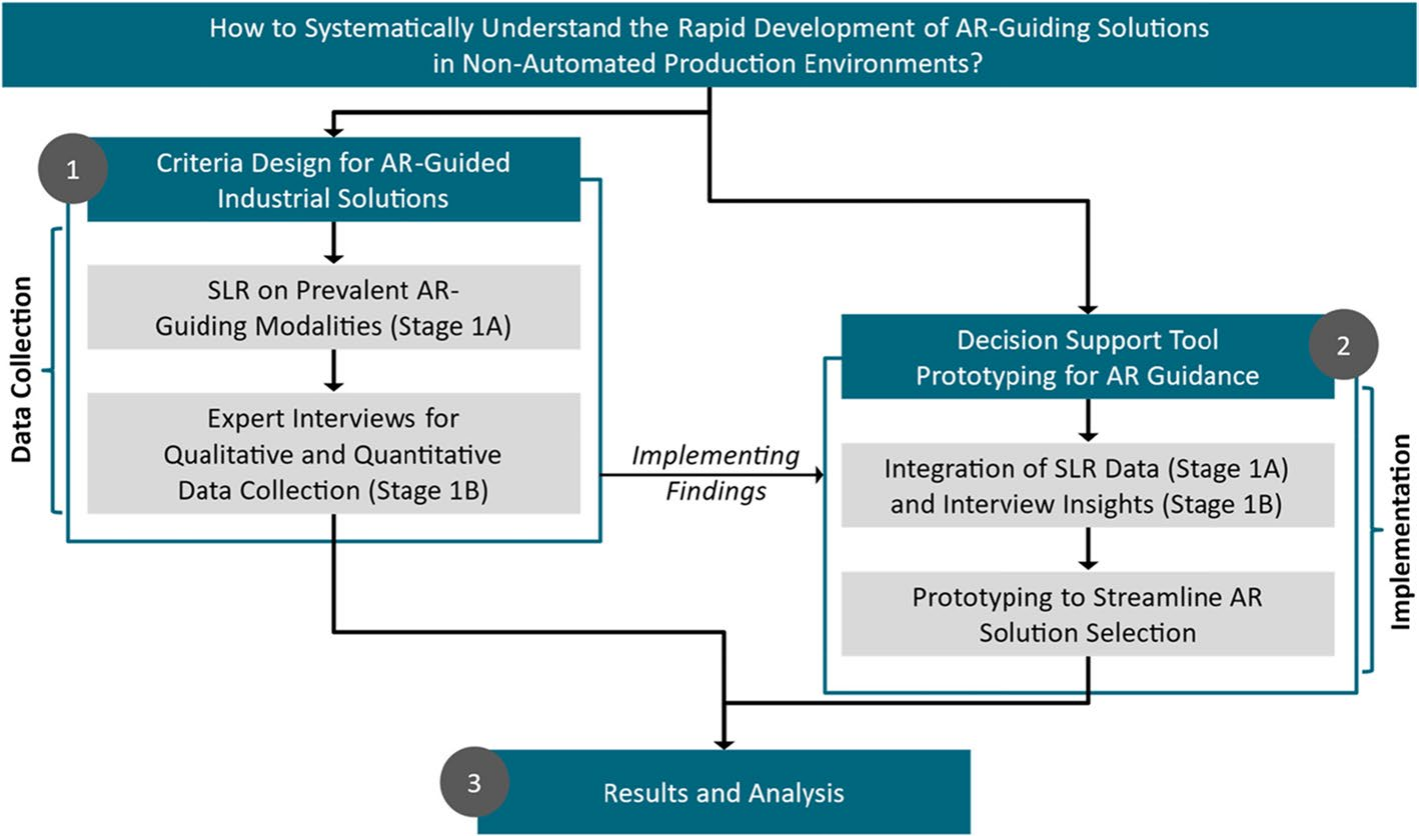
The diagram shows our three-stage methodology

To that end, we investigated in our paper what considerations have to be made when deploying AR-based assistance systems in industrial settings. Our work resulted in the development of an expert system that can serve as a tool to support the decision-making process concerning AR system deployment. Our exploration follows a three-stage approach as shown in Fig. 2. The reminder of the paper is structured as follows. In the following section we described our three-stage methodology based on the work by Loizeau et al. [25] (see Fig. 1). Next we discussed the approach to and results of systematic literature review (SLR) carried out as part of this process (Stage 1A). Here, we focused on the application and impact of AR-assisted manual assembly on production lines. This is followed by description of the expert interview carried out in order to collect further qualitative and quantitative data about AR usage in assembly, based on the complexity of the engineering asset being assembled (Stage 1B). The results of this first stage are then analysed and integrated for the development of our *Decision Support Tool* (Stage 2). Finally, in the reminder of the paper we present and discuss our overall results and findings (Stage 3).

## Methodology

As observed by Mota et al. [15], one of the significant challenges facing the development of assistance systems is the creation of effective and efficient instructions for operators. Traditional technical documentation often relies on a combination of text and drawings to convey information provided through paper-based, non-interactive manuals, requiring a high mental workload to comprehend fragmented instructions [16, 17]. On the other hand, AR offers advantages over these traditional methods by allowing for an interactive and immersive experience, which can help reduce cognitive load [18]. We can also enrich the instructions using various visual assets to convey the necessary information [10, 19]. However, this often requires the involvement of graphic and user experience designers, as the choice of visual assets must consider factors such as the targeted users, surroundings, digital environments, and the processes being performed with the help of AR [20]. Failure to properly consider these factors can lead to the inclusion of inappropriate visual assets, which in turn can result in decreased performance [21].

Furthermore, the need for research investigating the industrial deployment of AR-based systems has also been observed by other researchers who identified the need for rigorous grounding using domain expert evaluation [22–24].

**Fig. 3:**
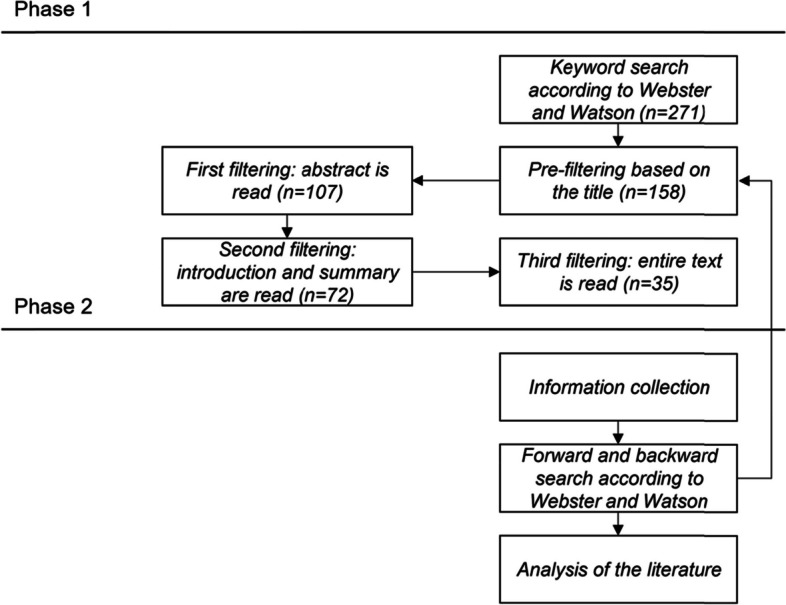
The diagram shows phases of our literature review process

Consequently, we adopted a three-stage process by Loizeau et al. [25], that ensures a systematic and informed approach to implementing AR in manual assembly operations (see Fig. 2). This process involves evaluating potential tasks to be supported with the AR application, selecting the most suitable one based on complexity and benefits, and then deploying the AR system to aid these specific operations. The initial stage consisted of a SLR and in-depth domain expert interviews. The systematic literature examination revealed limited evidence regarding the evaluation criteria for design decisions and the development of AR-based assistance systems (see Fig. 3). To bridge this knowledge gap, we conducted sixteen interviews with domain experts. Our approach diverges from Palmarini et al. [22], who focus on a decision-making tool specifically for maintenance operations, as we apply our findings to a broader industrial context involving more direct expert contributions in the development of our solutions. Unlike Eswaran et al. [23], who examined the effectiveness of AR visualisation modes through user studies for specific tasks, we believe that our research leads to the development of a more comprehensive decision support tool thanks to the utilized 3-step approach that combines systematic literature review with knowledge captured through expert interviews. Similarly, our work contrasts with Moghaddam et al. [24], who conducted laboratory experiments to evaluate AR’s impacts on task efficiency and learning, focusing primarily on immediate task-based outcomes.

Our two-part complementary approach (see Fig. 2) allowed us to identify relevant decision-making criteria and strategies behind the design and deployment of AR-based assisting systems. In the second stage, we prototyped the *Decision Support Tool* for AR-based guidance. This tool incorporated data from the systematic literature review and expert feedback to streamline the selection of AR solutions. Lastly, we aggregated and analysed all collected data in the third and final stage, culminating in a tool that not only guides the selection of AR systems but also adapts to the complexity of tasks in varied industrial settings, thereby extending the practical applications and theoretical contributions of our research beyond those outlined in prior works [22–24].

## Stage 1A: Systematic literature review

In this section we provide the overview of our SLR methodology, focusing on the use and effectiveness of AR for manual assembly in production environments. WE present the methodology used to collect and analyse relevant prior research, outline the technological advances in AR assistive systems, and discusses the key findings and gaps identified in the literature.

### Review methodology

Our literature search strategy is based on the two-phase process proposed by Jahangirian et al. [26]. It is a method that systematically integrates comprehensive keyword searches with subsequent selective criteria for an exploration and identification of relevant literature. Figure 3 illustrates the systematic reduction of reviewed publications throughout our literature search strategy, from the initial broad keyword search (e.g. “AR” OR “Augmented Reality” AND < “guidance” OR”industrial deployment” >) to the final selection. This figure succinctly illustrates the transition between phases and emphasises the iterative filtering process that is critical to narrowing down to the most relevant studies. It underlines the structured approach behind our selection, ensuring the comprehensiveness and relevance of our literature review. In the first phase, we identified 271 publications through our keyword search, which were then refined through Webster & Watson’s forward and backward search techniques [27]. Thanks to the backward search we examined references from our original articles to identify relevant foundational work, while the forward search helped us to identify recent studies that cited these articles, capturing current developments in the field. Such an approach ensured the comprehensiveness of our review, seamlessly linking historical and contemporary research findings. Next, the research papers were subjected to a systematic evaluation and selection using filters, as recommended by Brocke et al. [28]. The final criterion, which involved a comprehensive review of the abstracts, graphs, and full texts, allowed us to distil the collection to 35 critical manuscripts that form the core of our systematic literature review.

Central to our investigation is the main research question: *how to systematically understand the rapid deployment process of AR-guiding solution in non-automated industrial environments?* This question stems from the observation of the continued reliance on human labour in manufacturing alongside rapid advances in information technology and industrial digitization [7]. To provide an answer to such question, we have to first understand what are the key considerations in selecting guidance technologies and information conveyance approaches for AR-based guidance.

Consequently, our selection of literature was aligned with these considerations. The inclusion criteria for the review were based on the relevance to our specified keywords related to the outlined research question. Conversely, we excluded clearly unrelated publications, that were outside the question scope, or lacked empirical evidence. Such an approach ensures that our review was systematic and focused only on high quality studies relevant to the rapid development of AR technology in manual industrial processes such as manual assembly. We mainly considered AR-guided manual assembly solutions as such tools have demonstrated significant potential in addressing the growing complexity of assembly tasks and the need for versatile human workers [29].

### Literature review limitations

Our review process encounters inherent limitations due to data non-equivalence, particularly across different use cases and technology complexities [30]. This discrepancy highlights the challenge of making direct comparisons a critical aspect of both qualitative and comparative research methodologies. Furthermore, despite using a systematic methodology to define search terms and consulting a wide range of databases to mitigate selection bias, the potential for misclassification remains [31]. The overlapping nature of management approaches in industrial systems, and the variable differentiation and comparison metrics that depend on the unique context of each manufacturing organisation, further complicate the classification and analysis process.

### Augmented reality assistant systems

Mechanical assembly operations have been of significant interest to AR researchers for at least two decades [32, 33]. AR technology enables the 2D and 3D projection of instructions into the operator’s field of view (FoV), enriched with, for example, animations and textual descriptions in real-time during an assembly process [34, 35].

Compared to traditional guiding methods, like paper-based work instructions, search time for relevant instructions can be reduced by AR-based systems [36]. Furthermore, AR technology enables the worker to concentrate on the task by rendering instruction elements in the immediate vicinity of the work area to guide workers to complete specific tasks [37].

The performance of various AR instructions guiding assembly operations has been explored in the existing literature. For example, Tang et al. [38] conducted a study comparing the performance of an AR system with three different types of guiding methods. These were (i) printed, (ii) digital on a screen, and (iii) spatially registered AR (SAR) instructions. In the latter case, virtual objects appeared anchored or aligned with specific physical locations or real-world objects. The authors assessed the effectiveness of each method by measuring the number of errors, mental workload, and completion time. They remarked that SAR instructions improved task accuracy and reduced mental workload compared to other modalities [38]. However, SAR had no significant time advantage compared to the other approaches [38]. Moreover, further research remarked that SAR and other AR-projection techniques were unsuitable for manual production environments due to the high installation and calibration costs, limited mobility and adaptability [39–41].

An *in-situ* projected AR instruction for displaying assembly instructions was proposed by Funk et al. [42]. In an experiment, participants were tasked to solve an abstract *Lego Duplo*-based assembly task and the projected instructions were compared with the most common approaches: baseline paper instructions, tablet instructions, and head-mounted display (HMD) instructions. The in-situ projected AR instructions have been shown to reduce localisation time and cognitive workload compared to HMD instructions. However, a range of limitations was also identified, such as the projection being bound to a specific workstation and displaying only 2D information [42].

Hou et al. [43] presented an animated AR system embedded in a workstation using monitors and a tracking webcam. The animated instructions could be visualised on the monitor following a predefined marker. To compare AR instructions with paper-based instructions, the authors conducted two experiments. The effects of two types of instructions on the learning curves were analysed, including examining the cognitive performance when using assembly instructions. The results positively impacted cognitive facilitation with the animated AR system. The participants made fewer errors with the projection-based AR system, had lower cognitive load and performed faster. In addition, the learning curve of novice workers was shortened [43].

Another study was carried out by Blattgerste et al. [44], who developed instructions for the Microsoft HoloLens HMD and investigated the effects of 2D and 3D visualisations. They found that in-situ guiding with an HMD is advantageous compared to side-by-side guiding regarding error rate, task completion time, and task load. Furthermore, static 3D visualisation outperformed 2D and 3D instructions with wire-frames [44].

Werrlich et al. [17] investigated the training transfer of an HMD AR guiding system. For this purpose, they developed a multi-modal learning system that could be operated from the Microsoft HoloLens. The system was compared with a paper-based guide for assembling a car engine. The authors showed that HMD-based AR guiding leads to fewer errors and higher user satisfaction than paper-based instructions, although completion times were longer [17].

Moreover, similarly to other immersive technologies, the AR interface has to be populated with the appropriate 2D/3D, textual, audio, or other content in order to facilitate the instructions conveyance [45]. A critical facet in the AR assembly implementation is user interaction, demanding an intuitive interface that delineates the target functionality, such as guiding ongoing manual tasks [46]. In that context, we could see examples where researchers explored algorithms to automatically generate corresponding AR montage sequences [36]. In other cases, the instructions have to be previously captured through video and audio recordings in conjunction with the expert-knowledge capture and digitisation processes [45]. In the industrial scenario, often 3D content in the form of computer-aided design (CAD) models of work equipment and other work pieces is usually already available for use in the AR system [34, 35].

### Literature review results

Based on our systematic literature review, we observe that there is a research gap concerning preparing and implementing AR-guiding solutions across diverse production settings. This gap is particularly evident in decision making processes for selecting the most suitable AR (or non-AR) guiding approach. Numerous factors must be considered when designing, developing, and ultimately deploying a robust and effective solution. Consequently, there is a need for a deeper understanding of the deployment process for AR assistant systems, allowing us to navigate the complexities and challenges associated with integrating AR technology in various production environments [22]. In that context, through the systematic literature review, we identified a range of criteria and modalities that have to be considered when deciding on a particular AR guiding solution. These are listed in Table 1.

**Table 1:** Literature-informed list of criteria and modalities for deciding on an AR guiding solution

	Criterion	Description	References
1	Effort to obtain data	The effort required to gather essential data (e.g. textual instructions, labelled images, animations) for creating AR content	[27, 39, 47–51]
2	Error reduction	Assesses the assistance system's effectiveness in aiding workers to reduce error occurrences	[39, 52–59]
3	Completion time	Measures the impact of the assistance system in accelerating task completion by the worker	[39, 52–63]
4	Transferability	The resources and time needed to adapt the system for new tasks or processes	[64–68]
	Modality		
1	Text	Presented textual information. Here, the instruction clarity is critical to prevent confusion and delays	[47, 50, 56, 69]
2	Labelled picture	Use of images and annotated views to show component relationships, emphasising label clarity and colour coding	[52, 62, 69, 70]
3	Audio	Verbal instructions, offering a hands-free option that can complement or replace visual guides	[42, 50, 71]
4	Computer animation	Collision-free animations for complex assemblies, suitable for repeated viewings to clarify processes	[48, 49, 52, 69, 72]
5	Video	Video clips, showing real-time actions or graphical elements for enhanced clarity	[50, 53, 56–58, 69, 73]
6	Interactive system	Integrates systems responses to gesture, movement, body part tracking, object recognition etc. for an interactive instructional experience	[46, 47, 52, 74–76]

## Stage 1B: Expert interviews

In this section we detail the process of gathering data through the expert interviews, including the selection of participants from key technology fields, the structured interview design, and the analysis of results, highlighting emerging themes from industry and academic insights.

### Expert participants

We identified interviewees using a critical case purposive, non-probabilistic sampling technique [77]. Thus, we limited the selection pool of potential participants based on volunteers’ expertise and knowledge relevance to our research on AR-assisted guiding systems. We identify the domain experts leveraging our Home Institute's network of international collaborators from industry and academia alike working in the broader realms of advanced digital technologies and manufacturing within the UK and abroad. We aimed to have an equal number of interviews with experts from industry and academia to gain a broader and deeper understanding of the subject as well as to assess the potential differences between the two backgrounds. We also remark that some academic experts already had limited industrial experience. We selected participants with an industry background from strategic and tactical roles encompassing various responsibilities influencing the direction and efficiency of manufacturing processes. More details on the interviewees can be seen in Table 2.

**Table 2:** List of expert interview participants

P#	Sector	Position	Sex	Age	Experience
1	Conglomerate	Production Manager	M	41	25 + yrs. in industry
2	Conglomerate	Strategic Lead	M	50	25 + yrs. in R&D in industry
3	Consumer Goods and Adhesives	Digital Transformation Manager	M	25	2.5 yrs. in industry
4	Chemical and Pharmaceutical	Early Stage Researcher	F	31	3 yrs. in academia and 3.5 yrs. industry research
5	Aerospace	Programme Quality Manager	M	31	4 yrs. in industry
6	3D Technology and Software	Vice President	M	52	25 + yrs. in industry
7	Manufacturing	Industrial Researcher	M	27	3 yrs. in academia and 2 yrs. industry research
8	AR/VR Tools	Industrial Researcher	M	29	2.5 yrs. in academia and 1 year in industry
9	Manufacturing	Industrial Researcher	M	30	4 yrs. in academia and 3.5 yrs. in industry
10	Manufacturing	Postdoctoral Researcher	M	30	4,5 yrs. in academia and 1 year in industry
11	Manufacturing	Research Group Leader	M	/	6.5 yrs. in academia
12	Manufacturing	Research Associate	M	32	5 yrs. in academia and 2 yrs. in industry
13	VR & Speech Analysis	Associate Professor	F	36	12 yrs. in academia
14	VR Unity Designer	Developer	M	39	10 + yrs. in industry
15	Manufacturing	Research Associate	F	26	3 yrs. in academia
16	XR Metaverse	CEO	F	54	25 + yrs. in industry

The interviewees were offered the opportunity to name their respective sexes based on self-identification, following the *Sex and Gender Equity in Research* (SAGER) guidelines [78]. This binary categorisation, while not capturing the full spectrum of gender identities, aligns with common demographic data collection practices. Our sample included four self-identified female and twelve male participants, as shown in Table 2. The study was conducted primarily within the help of representatives from the European manufacturing sector, with the majority of participants based or educated in the UK. The gender distribution in our sample aligns with UK postgraduate engineering and technology degrees and exceeds the national engineering workforce's female representation, which is under 20% in the UK.^1^ Therefore, our sample is in line with the general female to male ratio within the manufacturing sector.

Furthermore, informed consent was obtained before the commencement of the interviews, and the Ethics Committee of the Engineering Department, University of Cambridge, approved the whole study in November 2022.

The sample size for the interviews was determined by the principle of theoretical saturation [79], meaning that enough interviews were conducted until we did not observe new relevant information for the evaluation criteria and AR approaches. Consequently, we interviewed sixteen domain-expert participants, roughly half of whom had industrial backgrounds and half were academics.

### Experimental design and interview queries

To structure the interviews, we prepared a literature-informed sheet with a range of the most frequently encountered criteria. Moreover, to provide a non-technological baseline, in addition to criteria enlisted in Table 1), we included paper-based and on-screen manuals to serve as examples of “classical approaches”.

When trying to gain new insights into the manual assembly process, we have to consider the complexity of the assembly task, which in turn is related to an asset being assembled. Here, we can apply the complexity description of information theory to manual manufacturing processes, which results in categorisation based on the number and variety of steps and parts as well as entropy dimensions that relate to the degree of uncertainty before an event o [82]. In this context, complexity in operations can be categorised as based on the variety and number of steps and parts involved, and the predictability of the sequence:

- Low-complexity involves a low variety and number of steps and parts, all arranged in a predetermined order.
- Medium-complexity features a wider variety and greater number of steps and parts, which are more challenging for operators to retain in working memory, yet still follow a predefined order.
- High-complexity is characterized by a high number of different parts and steps, with a varying sequence and significant uncertainty.

In the first step, we provided participants with definitions of task complexity. We also decided that the interviewees should select an assembly asset with which they had prior experience to ensure a more accurate evaluation based on their familiarity with the product. Participants were then instructed to assess the assembly tasks' complexity in the context of these chosen assets, which were to be of medium or high complexity. The list of use cases considered by domain experts during the interviews can be found in Table 3. This assessment ratings directly calibrate the operational parameters of our decision support tool, ensuring it adjusts its evaluations and recommendations based on the complexity levels documented in Table 3. Subsequent to this assessment, participants were asked to rate the effort required to obtain/generate necessary data such as instructions, pictures or technical drawings. They were also asked to rate the extent to which different guiding approaches could reduce errors during the task execution and the time at which the task could be completed using an AR system. These ratings, which reflect the error-proneness of each approach, directly influence the tool's evaluation metrics, prioritising methods that effectively minimise errors.

**Table 3:** Selected use cases and corresponding complexity

Use Case	Complexity	Description
Inverter Drive	Medium	Variable-size motor component assembly
Sensor Positioning	Medium	F1 car sensor placement on floors
Replacing Car Engine	High	Comprehensive car engine replacement process
Solar Panel for Satellites	High	Cabling for satellite solar panels
Engine	High	Detailed car engine repair process
Pneumatic Cylinder	Medium	Assembly of a multi-part cylinder
Converter	Medium	Electrical converter system assembly
RC Car	Medium	Assembly of diverse RC car models
Food Processor	Medium	Assembly of kitchen appliances (e.g. Siemens)
Gears	High	Gear assembly with over 20 variants
Engine	Medium	Mobility sector engine assembly
Machine Setting	Medium	Standardized machine setting guidance for operators
Filling up Container	Medium	Manual process for filling and sealing large containers
AR Goggles	High	Consumer AR goggles assembly

In the second step, we gave the participants the opportunity to enter additional items. We also tasked them with distributing 100 weight points between the criteria. We chose this approach to enable users to better perceive the relative importance of each factor with respect to the other guiding approaches [80].

When participants entered a value into a spreadsheet, the sum of all values was automatically calculated to make the selection process easier. We included the distributed weighting system to compel the interviewees to decide on the most important criteria.

In the final step, users were prompted to assign a score to each approach and the respective criterion related to it, using the Likert-like scale [81].

### Interview study results

In order to capture and analyse expert knowledge of the sixteen domain experts, we employed a detailed thematic analysis within a structured three-step method, which included data reduction, display, and conclusion [83]. We utilised NVivo 12 Plus to manage and categorise the qualitative data. During the data reduction phase, we identified key themes such as”technology”, “risks”, “criteria” and “decision-making”. This involved sifting through the data to extract pertinent segments that were relevant for focused examination. In the data display phase, we revisited the coding nodes, refining and reorganising them to ensure they accurately reflected overarching themes. This refinement was conducted iteratively to integrate and compare findings with existing literature, enhancing the depth of our analysis. The final phase, drawing conclusions, synthesised these findings into meaningful interpretations without emphasising frequency. In the analysis, six participants ascertained the assembly task as a high-complexity, and a medium-complexity use case was named in the remaining nine interviews. Table 3 contains the use cases with their respective complexity ratings as assessed by expert participants.

During these discussions, safety emerged as a critical criterion, as emphasised by the first participant with over two-and-half-decade experience under his belt who has made a strong case towards including such a parameter. This point was also raised by several other interviews domain experts (P1, P5, P7, P8, and P9; see Table 2). Consequently, we have added safety to our list of criteria as it was raised by the first participant, thus our interview process could be amended without compromising gathered data. Additionally, we considered the gearbox object (see Fig. 1(a)) as a low-complexity use case. For each complexity level, the data was aggregated, and a separate rating was made for each criterion. We informed participants that our study targets scenarios relevant to novice workers encountering new to them use cases, specifically products or their variants previously unfamiliar to them. This approach was intended not for creating a specific industrial guidance system but to assess how instructional designs can accommodate beginners’ learning needs.

Participants distributed a total of 100 points across the weights of each criterion, allowing a percentage-based perception of the relative importance of each criterion, as suggested by Shehabuddeen et al. [80]. We calculated the arithmetic mean of these weights per criterion for each level of complexity. To align the mean weights with the original 100-point scale, we normalised them so that their sum equalled 100. This normalisation was done using a standard scaling method where each mean weight was divided by the sum of all mean weights and then multiplied by 100. The resulting adjusted weights were then used to calculate the final scores for the different AR approaches by summing the product of these weights with the respective criterion scores.

**Fig. 4:**
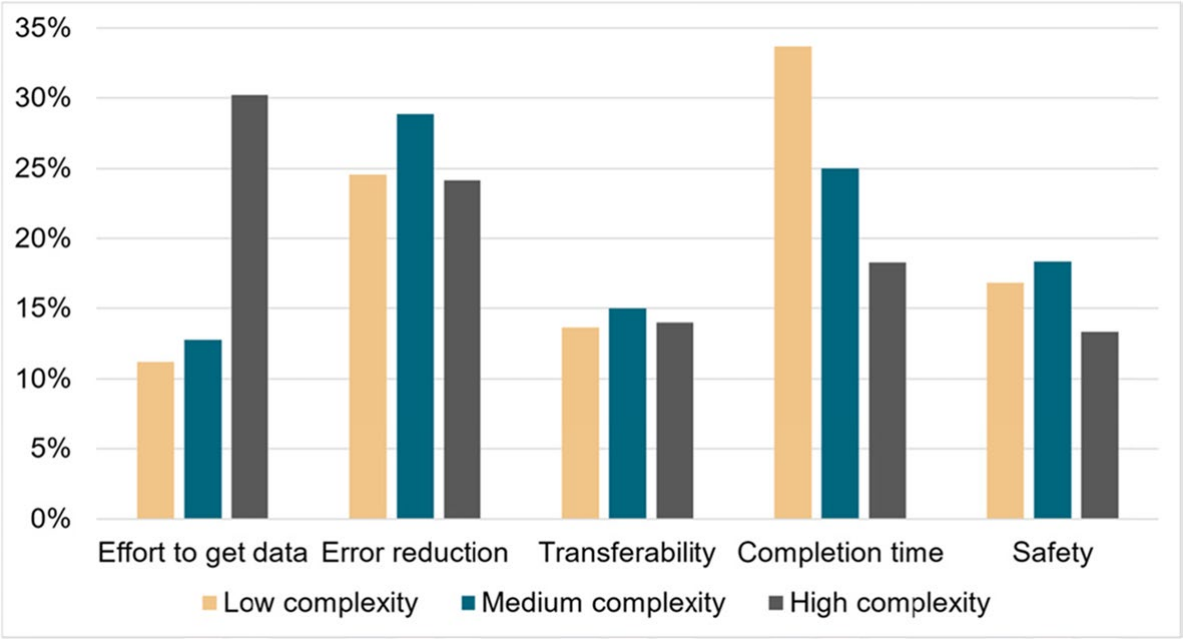
The graph shows the final score for different levels of complexity (low, medium, high) and guiding criteria

**Fig. 5:**
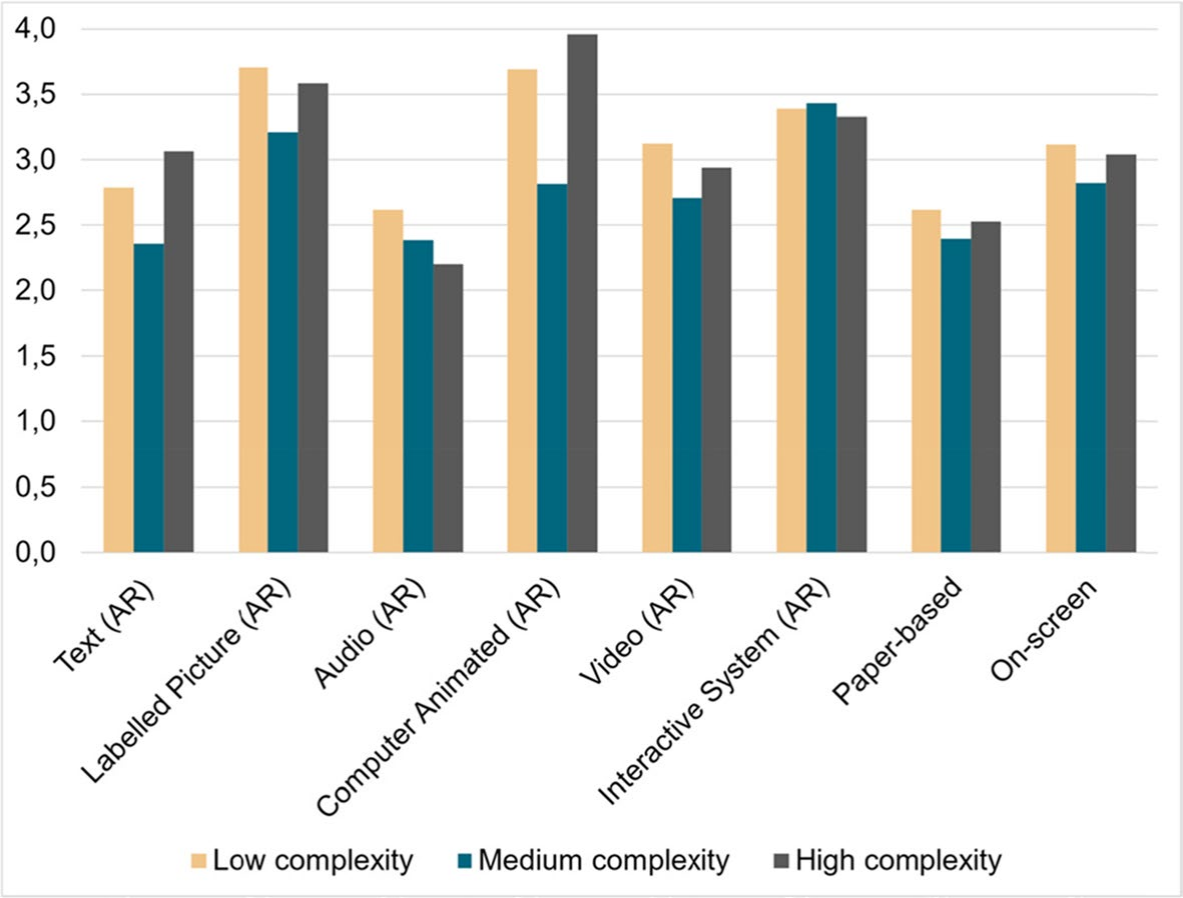
The graph shows the final score for different levels of complexity (low, medium, high) and guiding modalities

The mean values of the adjusted weights for the different complexities and criteria can be seen in Fig. 4. The resulting scores for the different complexities and guiding approaches can be seen in Fig. 5. Our main findings show that high complexity tasks have a three times higher weighting for the *effort to obtain data* (over 30%), due to the need for more detailed and precise instructions and more comprehensive data. Moreover, the integration of this data into usable AR content is typically more labour intensive, requiring extensive testing and iteration to ensure accuracy and usability. In contrast, the weightings for *error reduction* and *transferability* remain more consistent across complexity levels at 24–28% and around 15% respectively.

The *completion time* has the highest weighting for low-complexity tasks, i.e., almost 35%, and the lowest for high-complexity, i.e., 17%. This is consistent with the experts' explanations in the interviews, where for simpler tasks, time to completion is prioritised due to higher production volumes, whereas for complex tasks, quality is prioritised over time to completion. The safety criterion has the highest weighting for medium complexity tasks, i.e., 18%, and the lowest for high complexity tasks at 13%. Safety was particularly emphasised by industry experts. For medium complexity tasks, which may not have the inherent safeguards of high complexity environments but still present significant risks, the importance on safety is increased to mitigate potential hazards. In contrast, high complexity tasks often occur in environments where stringent safety protocols are already in place, so the incremental benefit of AR guidance on safety is perceived as less significant.

We observed no irregularities or strong disagreements within the data. The quantitative interview results for the low, medium and high-complexity scenarios can be seen in Table 4.

**Table 4:** Interview results for low, medium and high complexity

	Augmented Reality Assistance System						Classical Approaches	
Complexity	Text	Labelled Picture	Audio	Computer Animated	Video	Interactive System	Paper-based	On-screen
Low	2,8	3,7	2,6	3,7	3,1	3,4	2,6	3,1
Medium	2,4	3,2	2,4	2,8	2,7	3,4	2,4	2,8
High	3,1	3,6	2,2	4	2,9	3,3	2,5	3

### Expert interviews limitations

Our interview process involved sixteen domain experts with a range of industrial and academic backgrounds at various stages of their careers (see Table 2). Moreover, the ratio of male to female participants reflects on the gender distribution across the relevant manufacturing sector. Nevertheless, the sample size may not adequately represent all the views and expertise available in the manufacturing industry.

## Stage 2: Decision-support tool

**Fig. 6:**
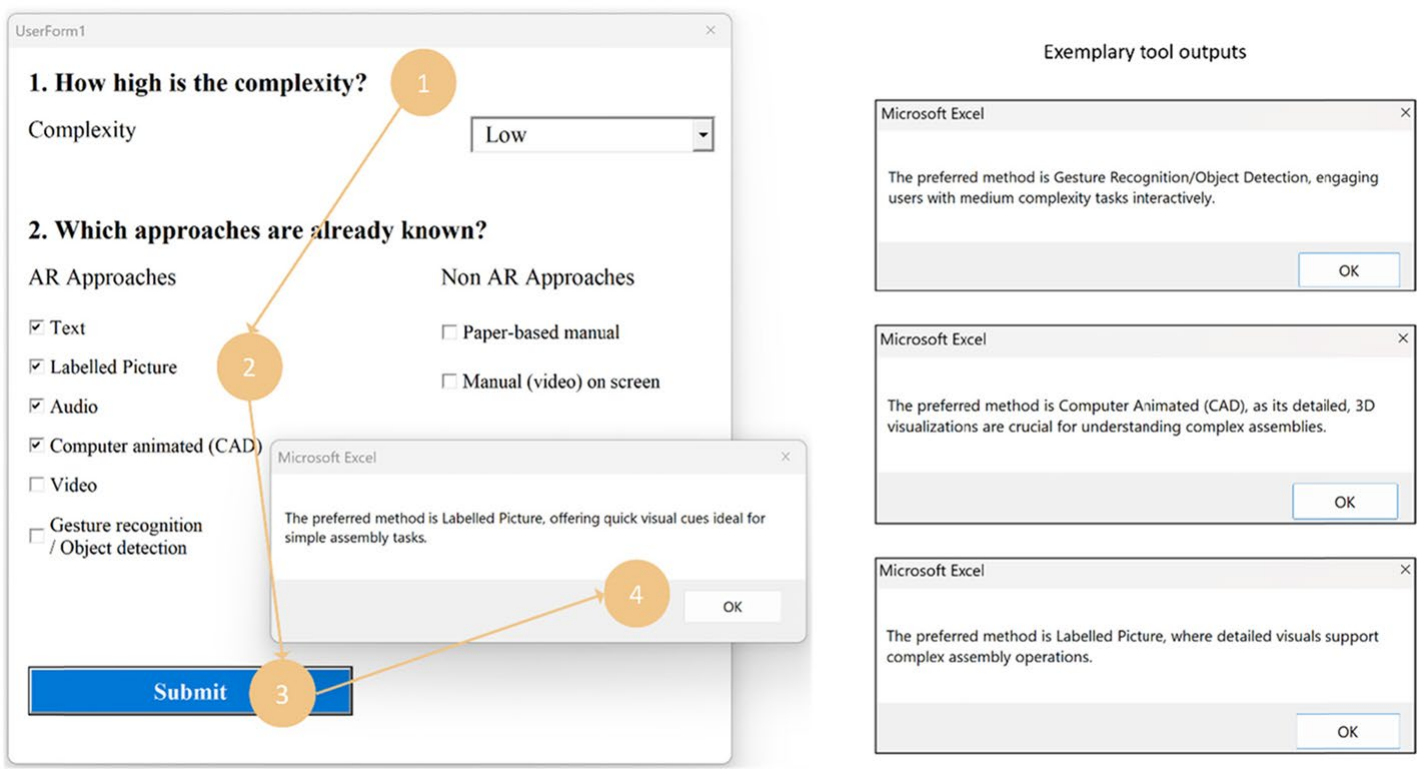
The screenshot of the selection support tool: (1) selection menu of the task complexity level, (2) selection menu for different AR-guiding modalities, (3) submit button, (4) pop-up textbox with suggested AR-guiding modality based on weights in Table 4. The right-hand side includes exemplary outputs on the right, demonstrating alternative recommended modalities for tasks of varying complexities

All the data we collected in the first stage through systematic literature review and domain-expert interviews (see Fig. 2 were used to prepare our *decision support tool* (see Fig. 6). As such, the tool incorporates all the identified modalities and crucial criteria needed to be observed when working towards deployment of an AR solution in an industrial context.

We developed our decision support tool utilising Microsoft’s Visual Basic for Applications (VBA) programming environment. We chose this approach as it allowed quick prototyping, testing and porting of experts-scoring already captured in an Excel spreadsheet and CSV files. If needed, the developer was able to promptly extend the tool with new weights and other data. In agreement with the open science movement [84], we made the collected data and the tool itself freely available as supplementary material to this manuscript.^2^

To use the tool, the user first needs to select the desired level of object complexity from a dropdown menu. The user is then prompted to specify which AR-based or non-AR-based approaches are available for consideration. This input allows the tool to consider the user provided information. Finally, the user needs to click the [*Submit*] button to obtain a recommendation based on the provided input and internally stored data.

## Stage 3: Discussion

In here we analyse and discuss the selection and implementation of AR-based assistance systems, focusing on the criteria necessary for effective adoption as well as the guiding modalities that influence user acceptance and system performance.

### Criteria and considerations for assistance aystem

Choosing a suitable, fit-for-purpose solution can be challenging as specific criteria for technology selection vary depending on the organisation’s needs and tasks that have to be supported [80]. To gain a deeper understanding and new insights into a more systematic selection process, we developed a literature-informed list of criteria and modalities most frequently considered in the design and deployment phases of AR-based assistance systems (see Table 1). We then presented this list of criteria to sixteen domain expert participants during an interview study (see Table 2). Here, one of the most frequently mentioned criteria in the interviews were *safety*, *user acceptance* and *usability*.

In this context, safety is defined not only as general workplace safety, but also as the ability of the AR system to alert users to potential hazards, provide preventive protection and, if necessary, intervene or stop operations if operator’s safety is compromised.

Interestingly, we observed a discrepancy between comments from academia and industry, with almost exclusively the experts with industrial backgrounds emphasising the *safety* criterion as crucial. This can be tentatively explained by the fact that academic user studies are typically carried out in safe, controlled laboratory spaces, under constant supervision and with relevant ethical approval from external bodies overseeing the studies.

Furthermore, experts identified key barriers to AR adoption in customised manufacturing, i.e., the need for extensive system reconfiguration due to product customisation and user resistance to new technologies. These challenges highlight the need for adaptable, user-friendly tools to support decision-making process related to AR deployment to encourage or discourage its adoption in a given industrial setting.

### AR Guiding modalities for assistance system

Choosing which components to implement in an assistive system, such as visual indicators, audio cues, and haptic feedback, plays a crucial role in determining system acceptance [1]. Earlier studies reflected that increased information acceptance had a statistically significant positive effect on error rate and efficiency [63]. Furthermore, according to scientific literature [85], as well as the results of our interviews, user acceptance is crucial to success. A recent study also highlighted the importance of evaluating AR acceptance in assembly work scenarios to ensure its effective utilisation [86].

We selected the approaches for the AR guiding systems based on the frequency of their reference in the literature (see Table 4). These modalities were evaluated and discussed during the expert interview study to ascertain their effectiveness and applicability. Here, participants’ main concern was that the chosen items (e.g. text, audio, or video) could be seen as too generic and largely dependent on their final implementation. Also, six interviewees remarked that the content to be displayed in AR depends on the complexity of the task and the individual user characteristics. Thus, hinting at the need for more person-optimised system rather than a general solution. Consequently, when completing our results, we informed the interview participants that the study assumes novice workers and a new use case scenario.

In this context, earlier research suggests that the impact of AR on mental workload and task performance varies based on user characteristics such as age demographics [87], domain expertise [70, 88], and familiarity with AR technology [89].

Expert Interview participants also highlighted the subjective and contextual nature of perceived complexity, illustrated by different industry requirements. For example, interview participant P5 observed that a task deemed low complexity in aviation may be considered high complexity in automotive, underscoring the variability across sectors. This example aligns with findings by Mattsson [90], who noted that workers' perceptions of complexity could vary significantly even within the same system, influenced by their expertise and the task context.

This underlines the necessity for a decision support tool that can guide the deployment of AR-based assistance systems in a manner tailored to the specific needs of the company and the industrial setting, ensuring both workplace acceptance and economic viability.

Additionally, when making final decisions, we must consider available skill sets among the development team. In this context, Table 5 synthesises the key recommendations for designing and deploying AR-based assistance systems, leveraging insights from the SLR and carrying out expert interviews. Each entry specifies a particular guideline and explains the rationale behind it and its potential implications for the user. Furthermore, our *Decision Support Tool* integrates these considerations, as evidenced in the outputs displayed in Fig. 6. This structured approach not only serves as a practical reference but also supports informed decision-making, crucial for effectively integrating AR technologies in typical production environments and managerial processes.

**Table 5:** Guidelines for designing and deploying AR-based assistance systems

Guideline	Recommendation	Rationale	Potential Implications
Hands-Free Operation	Ensure the AR system can be operated without manual handling	Enhances user safety and efficiency by allowing users to work with both hands on their tasks	Increased productivity and reduction in training time
Workstation Adaptability	The system should adapt to different workstation layouts	Accommodates various physical environments, enhancing flexibility and usability	Broader application across different tasks and settings
User-Friendliness	The interface should be intuitive for novices but adaptable for experienced users	Facilitates ease of use regardless of the user's prior experience with AR technology	Enhances the system's adoption rate and user satisfaction
Immersive Experience	Strive for a fully immersive user interaction within the assembly process	Increases engagement and accuracy in task execution	May lead to higher quality outputs and fewer errors
Task Statistics Recording	Integrate features for recording task data for future reference and planning	Assists in the continuous improvement of task strategies and resource allocation	Provides data-driven insights for optimizing future tasks
Documentation Integration	Allow users to add to existing documentation via multimedia inputs during tasks	Enhances the detail and accuracy of operational records and troubleshooting	Improves knowledge management and operational continuity
High Computational Power	Equip the system with powerful processing units to prevent lag	Ensures smooth, real-time operation, crucial for maintaining user engagement and task efficiency	Reduces frustrations and disruptions during critical tasks
High-Resolution Display and Audio	Include high-resolution visuals and clear audio output	Ensures clarity of information, which is critical for precision tasks and safety	Enhances user understanding and compliance with task requirements
Robust Connectivity	Maintain strong connection capabilities for data and documentation sync	Essential for real-time data sharing and collaboration across platforms	Ensures seamless integration and continuous workflow
Camera and CV Support	Incorporate high-quality cameras to support computer vision algorithms	Critical for detailed visual data capture and real-time decision-making support	Enhances the accuracy and adaptability of the system to complex tasks
Environmental and Safety Compatibility	Ensure compatibility with standard safety gear and regulations	Complies with workplace safety standards and reduces liability	Promotes safety and user acceptance in regulated environments
Glove-Friendly Operation	Design interfaces to work effectively with safety gloves	Necessary in environments requiring protective gear, addressing reduced touch sensitivity	Ensures the system remains functional and accessible in all required settings
Hands-Free Operation	Ensure the AR system can be operated without manual handling	Enhances user safety and efficiency by allowing users to work with both hands on their tasks	Increased productivity and reduction in training time

## Conclusion

AR assistance systems can support shop floor workers in manual tasks by facilitating seamless bidirectional information exchange, enhancing productivity and increasing work comfort [12]. This allows workers to focus on the value-adding aspects of their tasks while the assistance system handles most information processing. However, to develop and design such a system, we must first consider numerous technology-related and non-technical factors under a set of criteria and limitations. These, in turn, can be environmental, economic or technological in nature.

We addressed this need by identifying and assessing the key criteria for selecting an AR-based assistance system and different guiding modalities (see Table 4). We applied a three-stage approach [25] (see Fig. 2), involving a systematic literature review (see Fig. 3), subsequent expert-knowledge capture interview process (see Table 2), and analysis of the resulting data and observations. Our data-driven methodology contrasts and vastly extends prior approaches and findings and answers the need of grounding the research in expert domain knowledge [24].

Through this process (see Fig. 3), we synthesised and refined lists of criteria and modalities essential for making informed decisions on assistance deployment (see Table 1). Next, this set of criteria has undergone scrutiny in a literature-informed interview study with sixteen domain experts (see Table 2). Using the resulting data, we developed a Microsoft’s *Excel*-based VBA demonstrator as a decision support tool (see Fig. 6) that integrates the quantitative data to provide a systematic, data-driven guide for selecting an appropriate AR-based industrial control system aligned with the complexity levels and criteria validated by the experts. Our results highlight the importance of tailoring the design and deployment of AR systems according to the complexity of the tasks being performed, as different criteria gain varying levels of importance in other contexts.

In summary, our research integrates results into an easy-to-use expert system, offering a practical solution for developing and using AR-based assistive technologies in industrial settings. This approach allows the AR interface and assistance options to be customised to match the complexity of specific tasks and assets. By integrating a systematic review of existing literature and obtained domain expert feedback, we enable the customisation of AR interfaces to enhance decision-making and operational efficiency. Thus, empowering decision-makers to optimise worker experience and AR system performance. Additionally, our solution acts as a checklist for AR system deployment, providing guidance into what criteria and modalities are currently available when developing such an immersive interface.

## Future work

We plan to validate our decision support tool for selecting an appropriate AR-based guiding solution in a real-world setting with a range of industrial partners [80]. Furthermore, to extend its utility across various industries, bespoke modifications may prove to be necessary. To that end, we will engage with domain-specific experts through structured interviews to capture distinct operational requirements. These insights will be than integrated into our tool's algorithmic backend, enhancing its applicability and effectiveness in diverse settings. In addition to assessing the time constraints for deploying various AR systems, we will focus on developing personalised strategies that adjust AR interfaces and functionalities according to individual user characteristics. By leveraging empirical user data and advanced machine learning techniques, we will enhance our tool with information on how to dynamically adapt the AR environment to enhance user engagement and performance. This strategy will enable the AR system to adjust seamlessly to different product variants and manufacturing setups, ensuring scalability through continuous data-driven optimisation.

Moreover, we plan to also investigate the growing role of artificial intelligence in industrial processes, specifically, the use of computer vision for verification of instructions execution or object detection and tracking when coupled with an AR interface [12]. However, presently such approaches still present challenges that needs to be overcome before they could provide adequate support to the AR operators [12].

## Data Availability

We made the collected data and the tool itself freely available in the form of GitHub repository: https://github.com/skt40/DecisionSupportForAR.

## Footnotes

1 EngineeringUK, "Engineering in Higher Education," EngineeringUK, March 2023. [Online]. Available: https://www.engineeringuk.com/media/318874/engineering-in-higher-education_report_engineeringuk_march23_fv.pdf [Accessed: April 6, 2024].

2 https://github.com/skt40/DecisionSupportForAR

## References

[1] Moencks M, Roth E, Bohne T (2020) Cyber-physical operator assistance systems in industry: cross-hierarchical perspectives on augmenting human abilities. pp 419–423. 10.1109/IEEM45057.2020.9309734

[2] Schuh G, Gartzen T, Rodenhauser T, Marks A (2015) Promoting work-based learning through INDUSTRY 4.0. Procedia CIRP 32:82–87

[3] Jacobs FR, Chase RB, Lummus RR (2014) Operations and supply chain management. McGraw-Hill/Irwin, New York

[4] Lai Z-H, Tao W, Leu MC, Yin Z (2020) Smart augmented reality instructional system for mechanical assembly towards worker-centered intelligent manufacturing. J Manuf Syst 55:69–81. 10.1016/j.jmsy.2020.02.010

[5] Autor DH (2015) Why are there still so many jobs? The history and future of workplace automation. J Econ Perspect 29(3):3–30

[6] Baroroh DK, Chu C-H, Wang L (2021) Systematic literature review on augmented reality in smart manufacturing: collaboration between human and computational intelligence. J Manuf Syst 61:696–711. 10.1016/j.jmsy.2020.10.017

[7] Moencks M, Roth E, Bohné T, Kristensson PO (2022) Augmented workforce: contextual, cross-hierarchical enquiries on human-technology integration in industry. Comput Ind Eng 165:107822

[8] Moencks M, Roth E, Bohne T, Basso M, Betti F (2022) Augmented workforce: empowering people transforming manufacturing. https://www.weforum.org/whitepapers/augmented-workforce-empowering-people-transforming-manufacturing. Accessed 19 Jul 2024

[9] Bornewasser M, Bläsing D, Hinrichsen S (2018) Informatorische Assistenzsysteme in Der Manuellen Montage: Ein nützliches Werkzeug Zur Reduktion mentaler Beanspruchung? Z für Arbeitswissenschaft 72(4):264–275

[10] Tadeja SK, Solari Bozzi LO, Samson KDG, Pattinson SW, Bohné T (2023) Exploring the repair process of a 3D printer using augmented reality-based guidance, Computers & Graphics. 10.1016/j.cag.2023.10.017. https://www.sciencedirect.com/science/article/pii/S0097849323002546. Accessed 19 Jul 2024

[11] Eswaran M, Bahubalendruni MVAR (2022) Challenges and opportunities on AR/VR technologies for manufacturing systems in the context of industry 4.0: a state of the art review. J Manuf Syst 65:260–278. 10.1016/j.jmsy.2022.09.016

[12] Łysakowski M, Zywanowski K, Banaszczyk A, Nowicki MR, Skrzypczyński P, Tadeja SK (2023) Using AR and YOLOv8-based object detection to support real-world visual search in industrial workshop: lessons learned from a pilot study. In: 2023 IEEE International Symposium on Mixed and Augmented Reality Adjunct (ISMAR-Adjunct), pp 154–158. 10.1109/ISMAR-Adjunct60411.2023.00039

[13] Zhu J, Ong SK, Nee AYC (2012) An authorable context-aware augmented reality system to assist the maintenance technicians. Int J Adv Manuf Technol. 10.1007/s00170-012-4451-2

[14] Makris S, Pintzos G, Rentzos L, Chryssolouris G (2013) Assembly support using AR technology based on automatic sequence generation. CIRP Ann 62(1):9–12

[15] Khuong BM, Kiyokawa K, Miller A, La Viola JJ, Mashita T, Takemura H (2014) The effectiveness of an AR-based context-aware assembly support system in object assembly. In: 2014 IEEE virtual reality (VR). IEEE, pp 57–62. 10.1109/VR.2014.6802051

[16] Beetz S (2006) Beitrag Zur Methode Der Arbeitsplatz-Integrierten Assistenz am Beispiel Formmesstechnik. Vol. Bd. 14 of Berichte aus dem Lehrstuhl Qualitätsmanagement und Fertigungsmeßtechnik. Friedrich-Alexander-Universität Erlangen-Nürnberg, Shaker, Aachen

[17] Bengler K, Lock C, Teubner S, Reinhart G (2017) Grundlegende Konzepte Und Modelle. In: Reinhart G (ed) Handbuch Industrie 4.0. Carl Hanser Verlag, Hanser, München, Germany, pp 54

[18] Loizeau Q, Danglade F, Ababsa F, Merienne F (n.d.) Methodology for the field evaluation of the impact of augmented reality tools for maintenance workers in the aeronautic industry. https://www.frontiersin.org/articles/10.3389/frvir.2020.603189. Accessed 19 Jul 2024

[19] Mota RC, Roberto RA, Teichrieb V (2015) [POSTER] Authoring tools in augmented reality: an analysis and classification of content design tools. In: 2015 IEEE International Symposium on Mixed and Augmented Reality. IEEE, pp 164–167

[20] Henderson SJ, Feiner S (2009) Evaluating the benefits of augmented reality for task localization in maintenance of an armored personnel carrier turret. In: 2009 8th IEEE International Symposium on Mixed and Augmented Reality, IEEE, pp 135–144

[21] Werrlich S, Daniel A, Ginger A, Nguyen P-A, Notni G (2018) Comparing HMD-based and paper-based training. In: Proceedings of 2018 IEEE International Symposium on Mixed and Augmented Reality (ISMAR), Munich, Germany, pp 134–142. 10.1109/ISMAR.2018.00046

[22] Buchner J, Buntins K, Kerres M (2022) The impact of augmented reality on cognitive load and performance: a systematic review. J Comput Assist Learn 38(1):285–303. 10.1111/jcal.12617

[23] Gattullo M, Evangelista A, Uva AE, Fiorentino M, Gabbard JL (2022) What, how, and why are visual assets used in Industrial Augmented reality? A systematic review and classification in maintenance, assembly, and training (from 1997 to 2019). IEEE TVCG 28(2):1443–1456 32759085 10.1109/TVCG.2020.3014614

[24] Geng J, Song X, Pan Y, Tang J, Liu Y, Zhao D, Ma Y (2020) A systematic design method of adaptive augmented reality work instruction for complex industrial operations. Comput Ind 119:103229

[25] Tang YM, Au KM, Lau HCW, Ho GTS, Wu CH (2020) Evaluating the effectiveness of learning design with mixed reality (MR) in higher education. Virtual Reality 24(4):797–807

[26] Palmarini R, Erkoyuncu JA, Roy R (2017) An innovative process to select augmented reality (ar) technology for maintenance. Procedia CIRP 59:23–28. 10.1016/j.procir.2016.10.001. Proceedings of the 5th International Conference in Through-life Engineering Services Cranfield University, 1st and 2nd November 2016

[27] Eswaran M, Raju Bahubalendruni MVA (2023) Augmented reality aided object mapping for worker assistance/training in an industrial assembly context: exploration of affordance with existing guidance techniques. Comput Ind Eng 185:109663

[28] Moghaddam M, Wilson NC, Modestino AS, Jona K, Marsella SC (2021) Exploring augmented reality for worker assistance versus training. Adv Eng Inf 50:101410

[29] Jahangirian M, Eldabi T, Naseer A, Stergioulas LK, Young T (2010) Simulation in manufacturing and business: a review. Eur J Oper Res 203(1):1–13

[30] Webster J, Watson RT (2002) Analyzing the past to prepare for the future: writing a literature review. https://www.jstor.org/stable/4132319

[31] Brocke JV, Simons A, Niehaves B, Riemer K, Plattfaut R, Cleven A (2009) Reconstructing the giant: on the importance of rigour in documenting the literature search process. In: Proceedings of European Conference on Information Systems (ECIS), pp 161. https://aisel.aisnet.org/ecis2009/161

[32] Wang Z, Bai X, Zhang S, Wang Y, Han S, Zhang X, Yan Y, Xiong Z (2020) User-oriented ar assembly guideline: a new classification method of assembly instruction for user cognition. Int J Adv Manuf Technol 112:41–59

[33] Miri SM, Sharokh ZD (2019) A short introduction to comparative research. Allameh Tabataba'i University, Faculty of Management & Accounting, Department of Business Management, Tehran, Iran

[34] Moencks M, Roth E, Bohné T, Kristensson PO (2022) Human-computer interaction in industry: a systematic review on the applicability and value-added of operator assistance systems. In: Foundations and Trends^®^ in Human–Computer Interaction, vol 16, no 2–3, pp 65–213. 10.1561/1100000088

[35] Raghavan V, Molineros J, Sharma R (1999) Interactive evaluation of assembly sequences using augmented reality. IEEE Trans Robot Autom 15(3):435–449

[36] Sharma R, Molineros J (1997) Computer vision-based augmented reality for guiding manual assembly. Presence: Teleoperators and Virtual Environments 6(3):292–317

[37] Rentzos L, Papanastasiou S, Papakostas N, Chryssolouris G (2013) Augmented reality for human-based assembly: using product and process semantics. IFAC/IFIP/IFORS/IEA Symposium on Analysis, Design, and Evaluation of Human-Machine Systems. 10.3182/20130811-5-US-2037.00053

[38] Tang A, Owen C, Biocca F, Mou W (2003) Comparative effectiveness of augmented reality in object assembly. In: Cockton G, Korhonen P (eds) Proceedings of the conference on Human factors in computing systems - CHI ’03, ACM Press, pp 73. 10.1145/642611.642626

[39] Lavric T, Bricard E, Preda M, Zaharia T (2021) An industry-adapted AR training method for manual assembly operations. In: Stephanidis C et al (eds) HCI International 2021 - late breaking papers: multimodality, extended reality, and artificial intelligence. HCII 2021. Lecture Notes in Computer Science, vol 13095. Springer, Cham. 10.1007/978-3-030-90963-5_22

[40] Mengoni M, Ceccacci S, Generosi A, Leopardi A (2018) Spatial augmented reality: an application for human work in smart manufacturing environment. Procedia Manuf 17:476–483

[41] Uva AE, Gattullo M, Manghisi VM, Spagnulo D, Cascella GL, Fiorentino M (2018) Evaluating the effectiveness of spatial augmented reality in smart manufacturing: a solution for manual working stations. Int J Adv Manuf Technol 94(1–4):509–521

[42] Funk M, Heusler J, Akcay E, Weiland K, Schmidt S (2016) Haptic, auditory, or visual? towards optimal error feedback at manual assembly workplaces. In: Proceedings of the 9th ACM international conference on pervasive technologies related to assistive environments (PETRA '16). Association for Computing Machinery (ACM), New York, NY, USA, Article 43, pp 1–6. 10.1145/2910674.2910683

[43] Hou L, Wang X, Bernold L, Love PED (2013) Using animated augmented reality to cognitively guide assembly. J Comput Civil Eng 27(5):439–451

[44] Blattgerste J, Renner P, Strenge B, Pfeiffer T (2018) In-Situ Instructions Exceed Side-by-Side Instructions in AR Assisted Assembly. In: Proceedings of ACM PETRA, ACM, pp 133–140. 10.1145/3197768.3197778

[45] Bozzi LOS, Samson KDG, Tadeja S, Pattinson S, Bohné T (2023) Towards ar guiding systems: an engineering design of an immersive system for complex 3d printing repair process. In: 2023 IEEE Conference on Virtual Reality and 3D User Interfaces Abstracts and Workshops (VRW), pp 384–389. 10.1109/VRW58643.2023.00084

[46] Fang W, Hong J (2022) Bare-hand gesture occlusion-aware interactive augmented reality assembly. J Manuf Syst 65:169–179. 10.1016/j.jmsy.2022.09.009

[47] Begout P, Kubicki S, Bricard E, Duval T (2022) Augmented reality authoring of digital twins: design, implementation and evaluation in an industry 4.0 context. Front Virtual Real 3:918685. 10.3389/frvir.2022.918685

[48] Camba JD, Contero M (2015) From reality to augmented reality: rapid strategies for developing marker-based ar content using image capturing and authoring tools. In: Proceedings of the 2015 IEEE frontiers in Edu conference. IEEE Computer Society, USA, pp 1–6. 10.1109/FIE.2015.7344162

[49] Engelke T, Keil J, Rojtberg P, Wientapper F, Schmitt M, Bockholt U (2015) Content first: a concept for industrial augmented reality maintenance applications using mobile devices. In: Proceedings of the 6th ACM Multimedia Systems Conference, MMSys ’15, ACM, New York, pp 105–111

[50] Kong J, Sabha D, Bigham JP, Pavel A, Guo A (2021) Tutoriallens: authoring interactive augmented reality tutorials through narration and demonstration. In: Proceedings of the 2021 ACM Symposium on Spatial User Interaction, ACM, New York

[51] Chidambaram S, Huang H, He F, Qian X, Villanueva AM, Redick TS, Stuerzlinger W, Ramani K (2021) Processar: an augmented reality-based tool to create in-situ procedural 2d/3d ar instructions. In: Designing Interactive Systems Conference, ACM, ACM, Virtual Event, USA, p 16

[52] Wang X, Ong SK, Nee A (2016) Multi-modal augmented-reality assembly guidance based on bare-hand interface. Adv Eng Inf 30(3):406–421

[53] Lušic M, Fischer C, Bönig J, Hornfeck R, Franke J (2016) Worker Information systems: state of the art and guideline for selection under´ consideration of Company specific boundary conditions. Procedia CIRP 41:1113–1118

[54] Kim S, Nussbaum MA, Gabbard JL (2019) Influences of augmented reality head-worn display type and user interface design on performance and usability in simulated warehouse order picking. Appl Ergon 74:186–193 30487099 10.1016/j.apergo.2018.08.026

[55] Funk M, Bächler A, Bächler L, Korn O, Krieger C, Heidenreich T, Schmidt A (2015) Comparing projected in-situ feedback at the manual assembly workplace with impaired workers. In: Makedon F (ed) Proceedings of the ACM PETRA, ACM, pp 1–8. 10.1145/2769493.2769496

[56] Wang C-H, Tsai N-H, Lu J-M, Wang M-JJ (2019) Usability evaluation of an instructional application based on Google glass for mobile phone disassembly tasks. Appl Ergon 77:58–69. 10.1016/j.apergo.2019.01.007 30832779 10.1016/j.apergo.2019.01.007

[57] Lu Y, Mayol-Cuevas W (2021) The object at hand: automated editing for mixed reality video guidance from hand-object interactions. In: Proceedings of the IEEE International Symposium on Mixed and Augmented Reality (ISMAR), pp 90–98. 10.1109/ISMAR52148.2021.00023

[58] Cao Y, Fuste A, Heun V (2022) MobileTutAR: a lightweight augmented reality tutorial system using spatially situated human segmentation videos. In: Barbosa S, Lampe C, Appert C, Shamma DA (eds) CHI conference on human factors in computing systems extended abstracts. ACM, pp 1–8. 10.1145/3491101.3519639

[59] Türkmen R, Pfeuffer K, Barrera Machuca MD, Batmaz AU, Gellersen H (2022) Exploring discrete drawing guides to assist users in accurate mid-air sketching in VR. In: Extended Abstracts of the 2022 CHI Conference on Human Factors in Computing Systems, CHI EA ’22, ACM, New York. 10.1145/3491101.3519737

[60] Funk M, Kosch T, Greenwald SW, Schmidt A (2015) A benchmark for interactive augmented reality instructions for assembly tasks. In: Holzmann C, Mayrhofer R, Häkkilä J, Rukzio E, Roland M (eds) Proceedings of the 14th International Conference on Mobile and Ubiquitous Multimedia, ACM, pp 253–257

[61] Murauer N, Pflanz N, Hassel CV (2018) Comparison of scan-mechanisms in augmented reality-supported order picking processes. SmartObjects@CHI. https://api.semanticscholar.org/CorpusID:19216588. Accessed 19 Jul 2024

[62] Aehnelt M (2017) Informationsassistenz zur kognitiven automatisierung manueller Montagearbeitsplätze, Doctoral Thesis, Advisors: Urban B, Sandkuhl K, Lindstaedt S. University of Rostock

[63] Sochor R, Schick TS, Merkel L, Braunreuther S, Reinhart G (2020) Current knowledge management in manual assembly – further development by the analytical hierarchy process, incentive and cognitive assistance systems. In: Nyhuis P, Herberger D, Hübner M (eds) Proceedings of the Conference on Production Systems and Logistics: CPSL 2020, Publishing., Hannover, pp 209–219. 10.15488/9662

[64] Kozek M (2020) Transfer learning algorithm in image analysis with augmented reality headset for industry 4.0 technology. In: 2020 International MSM Conference, pp 1–5. 10.1109/MSM49833.2020.9201739

[65] Zhen W, Dunbing T, Changchun L, Xin X, Linqi Z, Zhuocheng Z, Xuan L (2021) Augmented-reality-assisted bearing fault diagnosis in intelligent manufacturing workshop using deep transfer learning. In: 2021 Global Reliability and Prognostics and Health Management (PHM-Nanjing), pp 1–6. 10.1109/PHM-Nanjing52125.2021.9613117

[66] Fernández del Amo I, Erkoyuncu JA, Roy R, Palmarini R, Onoufriou D (2018) A systematic review of augmented reality content-related techniques for knowledge transfer in maintenance applications. Comput Ind 103:47–71. 10.1016/j.compind.2018.08.007

[67] Lum WY, Lau F (2002) A context-aware decision engine for content adaptation. IEEE Pervasive Comput 1(3):41–49. 10.1109/MPRV.2002.1037721

[68] Jeske T, Meyer F, Schlick CM (2014) Einfluss Der Gestaltung Von Arbeitsplänen Auf die Anlernzeit Sensumotorischer Tätigkeiten. Z für Arbeitswissenschaft 68(1):1–6

[69] Chi H-L, Chen Y-C, Kang S-C, Hsieh S-H (2012) Development of UI for tele-operated cranes. Adv Eng Inf 26(3):641–652

[70] Fiorentino M, Uva AE, Gattullo M, Debernardis S, Monno G (2014) Augmented reality on large screen for interactive maintenance instructions. Comput Ind 65(2):270–278

[71] Laviola E, Gattullo M, Manghisi VM, Fiorentino M, Uva AE (2021) Minimal AR: visual asset optimization for the authoring of augmented reality work instructions in manufacturing. Int J Adv Manuf Technol 119(3–4):1769–1784 34866738 10.1007/s00170-021-08449-6 PMC8629731

[72] Leiva G, Nguyen C, Kazi RH, Asente P (2020) Pronto: rapid augmented reality video prototyping using sketches and enaction. In: Proceedings of CHI ’2, ACM, New York, pp 1–13

[73] Wang T, Qian X, He F, Hu X, Cao Y, Ramani K (2021) Gesturar: An authoring system for creating freehand interactive augmented reality applications. In: The 34th Annual ACM Symposium on User Interface Software and Technology, UIST ’21, ACM, New York, pp 552–567. 10.1145/3472749.3474769

[74] Chien C-H, Chen C-H, Jeng T-S (2010) An interactive augmented reality system for learning anatomy structure. In: Presented at the Proceedings of the International MultiConference of Engineers and Computer Scientists 2010, IMECS, pp 370–375

[75] Chen R, Tian X (2023) Gesture detection and recognition based on object detection in complex background. Appl Sci 13(7):4480. 10.3390/app13074480

[76] Saunders M, Lewis P, Thornhill A (2009) Research methods for Business Students. Pearson, New York

[77] Heidari S, Babor T, De Castro P et al (2016) Sex and gender equity in research: rationale for the sager guidelines and recommended use. Res Integr Peer Rev 1(2). 10.1186/s41073-016-0007-6 10.1186/s41073-016-0007-6 PMC5793986 29451543

[78] Glaser, BG, Strauss, AL (2017). Discovery of grounded theory: strategies for qualitative research, Routledge, New York. 10.4324/9780203793206

[79] Bendzioch S, Bläsing D, Hinrichsen S (2020) Comparison of different assembly assistance systems under ergonomic and economic aspects. In: Ahram T, Karwowski W, Pickl S, Taiar R (eds) Human systems engineering and design II, vol. 1026 of advances in intelligent systems and computing. Springer International Publishing, Cham, Switzerland, pp 20–25

[80] Shehabuddeen N, Probert D, Phaal R (2006) From theory to practice: challenges in operationalising a technology selection framework. Technovation 26(3):324–335

[81] Spector PE (1994) Using self-report questionnaires in ob research: a comment on the use of a controversial method. J Organizational Behav 15:385–392

[82] Miles MB, Huberman AM, Saldaña J (2014) Qualitative data analysis: a methods sourcebook, 3rd edn. Sage, Los Angeles

[83] Understanding open science - UNESCO digital library (2022) 10.54677/UTCD9302. https://unesdoc.unesco.org/ark:/48223/pf0000383323. Accessed 19 Jul 2024

[84] Masood T, Egger J (2019) Augmented reality in support of industry 4.0—Implementation challenges and success factors. Rob Computer- Integr Manuf 58:181–195

[85] Schuster F, Engelmann B, Sponholz U, Schmitt J (2021) Human acceptance evaluation of ar-assisted assembly scenarios. J Manuf Syst 61:660–672. 10.1016/j.jmsy.2020.12.012

[86] Kim S, Dey AK (2016) Augmenting human senses to improve the user experience in cars: applying augmented reality and haptics approaches to reduce cognitive distances. Multimedia Tools Appl 75(16):9587–9607

[87] Chalhoub J, Ayer SK (2019) Exploring the performance of an augmented reality application for construction layout tasks. Multimedia Tools Appl 78(24):35075–35098

[88] Stadler S, Kain K, Giuliani M, Mirnig N, Stollnberger G, Tscheligi M (2016) Augmented reality for industrial robot programmers: Workload analysis for task-based, augmented reality-supported robot control. In: 2016 25th IEEE International Symposium on Robot and Human Interactive Communication, pp 179–184. IEEE

[89] Mattsson S, Tarrar M, Fast-Berglund Å (2016) Perceived production complexity – understanding more than parts of a system. Int J Prod Res. 10.1080/00207543.2016.1154210

[90] Hoerner L, Schamberger M, Bodendorf F (2023) Using tacit expert knowledge to support shop-floor operators through a knowledge-based assistance system. CSCW 32(1):55–91

